# Supplementary material for: A Biomolecular Circuit for Automatic Gene Regulation in Mammalian Cells with CRISPR Technology
Source: ACS Synth Biol. 2024 Dec 2;13(12):3917–25. doi: 10.1021/acssynbio.4c00225 (PMC11669156; doi:10.1021/acssynbio.4c00225)
Supplement: Supplementary file 1 — sb4c00225_si_001.pdf [file sb4c00225_si_001.pdf]

Supporting Information

A biomolecular circuit for Automatic Gene Regulation in Mammalian Cells with CRISPR technology

Authors

Alessio Mallozzi[1,#,$], Virginia Fusco[1,2], Francesco Ragazzini[1,3], Diego di Bernardo[1,4,*]

Affiliations

[1] Telethon Institute of Genetics and Medicine, 80078, Naples, Italy

[2] University of Naples Federico II, Department of Electrical Engineering and Information Technologies, 80121, Naples, Italy

[3] Scuola Superiore Meridionale - School for Advanced Studies, 80138, Naples, Italy

[4] University of Naples Federico II, Department of Chemical Materials and Industrial Engineering, 80125, Naples,

Italy

[*] Correspondence should be addressed to D. di Bernardo: dibernardo@tigem.it

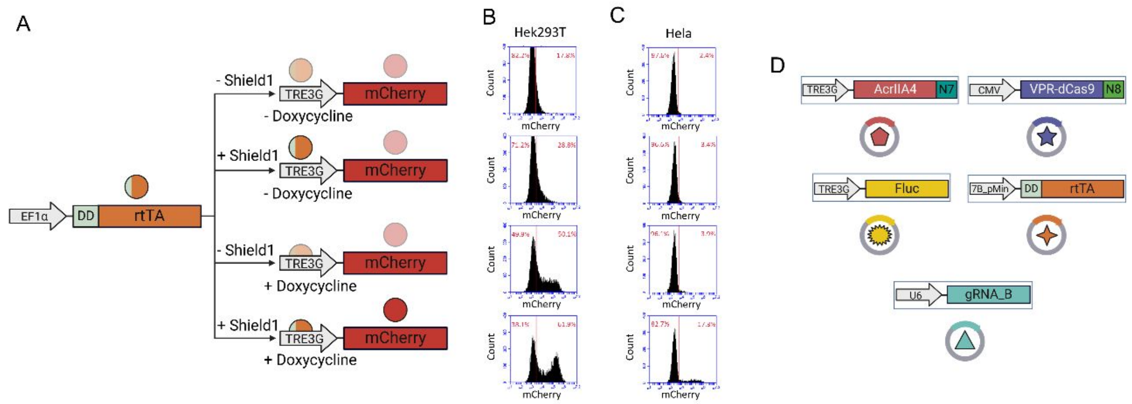

Figure S1 – Destabilization Domain and Dose-Reponse: A) Schematic representations of the experiment to test the correct function of the DD-rtTA fusion protein. DD-rtTA is constitutively expressed by the pEF1α promoter, while the pTRE3G promoter drives mCherry expression. In the absence of Doxycycline, DD-rtTA cannot bind pTRE3G promoter thus preventing mCherry transcription; in the absence of Shield1, DD-rtTA is unstable and quickly degraded. Only in the presence of both Shiel1 and Doxycycline, the mCherry can be fully expressed. **B, C)** mCherry fluorescence intensity distribution measured through flow cytometry in Hek293T cells (B) and in HeLa cells (C) transfected with pEF1α-DD-rtTA and pTRE3G-mCherry for the four corresponding conditions. **D)** Schematic representation of the five plasmids encoding for the CRISPRaTOR.

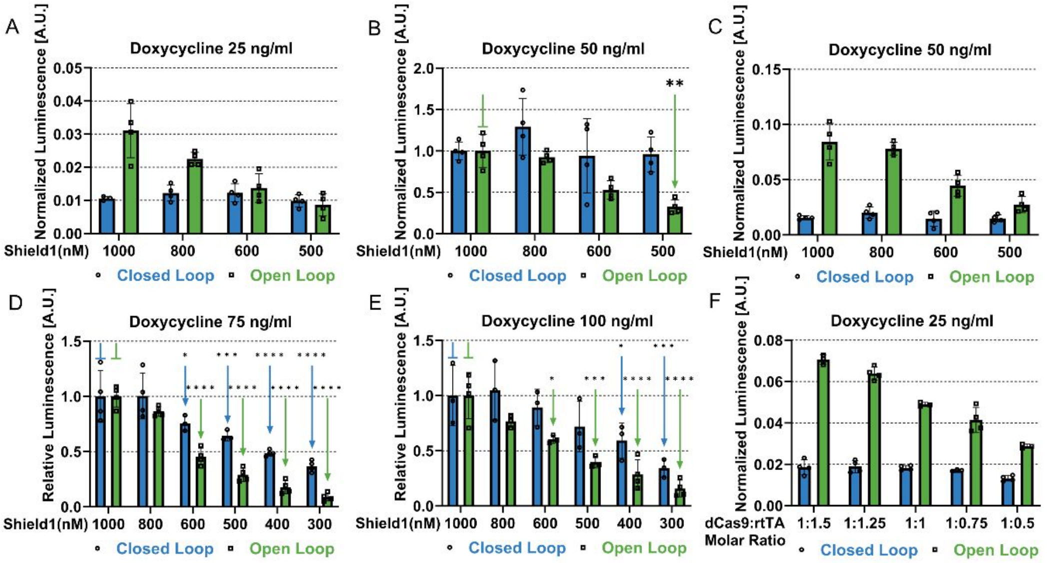

Figure S2 – Robust Perfect Adaptation in transient transfection in Hek293T cells. A) Normalized fLuc luminescence values for the experiment in Figure 1b. Normalised luminescence is measured as the fLuc luminescence normalised against Renilla luminescence. The concentration of doxycycline is kept constant at 25 ng/ml. **B)** Relative fLuc luminescence is computed as the normalized fLuc luminescence at the indicated concentrations of the Shield1 molecule, divided by its value at 1000 nM of Shield1. Doxycycline is kept constant at 50ng/ml. The green pointed arrows indicate significant difference in relative luminescence versus the value indicated by the green blunted arrow. **C)** Normalized luminescence of the fLuc reporter at the indicated concentrations of the Shield1 molecule. The concentration of doxycycline is kept constant at 50 ng/ml. **D, E)** Relative luminescence as in (B) for the indicated concentrations of doxycycline. The pointed arrows indicate a significant difference in relative luminescence versus the value indicated by the blunted arrow of the same color (blue or green). **F)** Normalized fLuc luminescence values for the experiment in Figure 1d. Normalised luminescence is measured as the fLuc luminescence normalised against Renilla luminescence at the indicated molar ratios. *n=4 biological replicates. A minimum of n=3 when one of the measurements was identified as an outlier (Grubbs' test, alpha=0.2). Statistics analysis has been conducted through a two-way ANOVA test. * $P \leq 0.05$ ** $P \leq 0.01$ *** $P \leq 0.001$ **** $P \leq 0.0001$.*

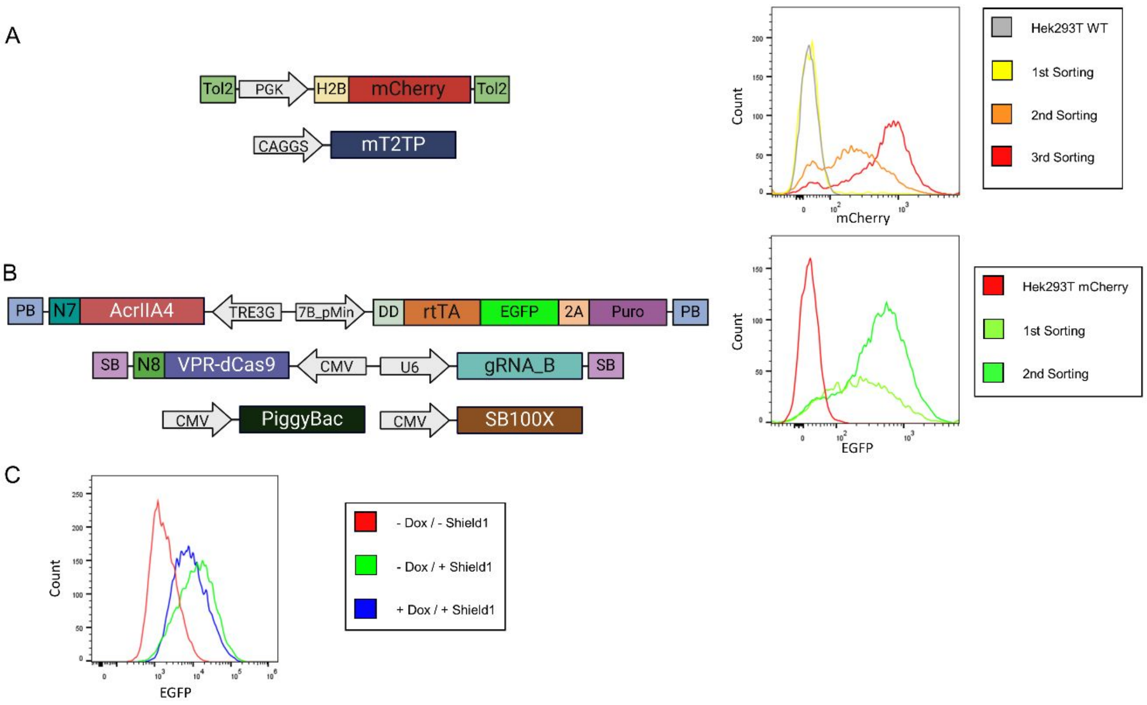

Figure S3 – Stable Cell Line Development: A) Results of cell sorting for red fluorescence with Fluorescence Activated Cell Sorter (FACS) after transfection of indicated plasmids. Both the percentage of mCherry-positive cells and the fluorescence intensity increase through the three cell sorting rounds. **B)** Results of cell sorting by FACS for green fluorescence after the transfection of the indicated plasmids in mCherry positive cells following cells expansion and Puromycin (1,5 µg/ml) selection. The percentage of EGFP -positive cells and the fluorescence intensity increase through the rounds of cell sorting, performed in the presence Shield1 (1000 ng/ml). **C)** Flow cytometry analysis of green fluorescence in Hek293T cells with genomic integration of the CRISPRaTOR. In absence of Shield1, DD-rtTA-EGFP is unstable and degraded. Upon addition of Shield1 (1000 nM), the protein is stabilized, and the cells exhibit a shift in the fluorescent signal. When also Doxycycline (1000 ng/ml) is added, DD-rtTA-EGFP can bind to the pTRE3G promoter and express AcrIIA4-N7 that inhibits VPR-dCas9-N8, thus closing feedback loop and resulting in a decrease in the DD-rtTA-EGFP expression.

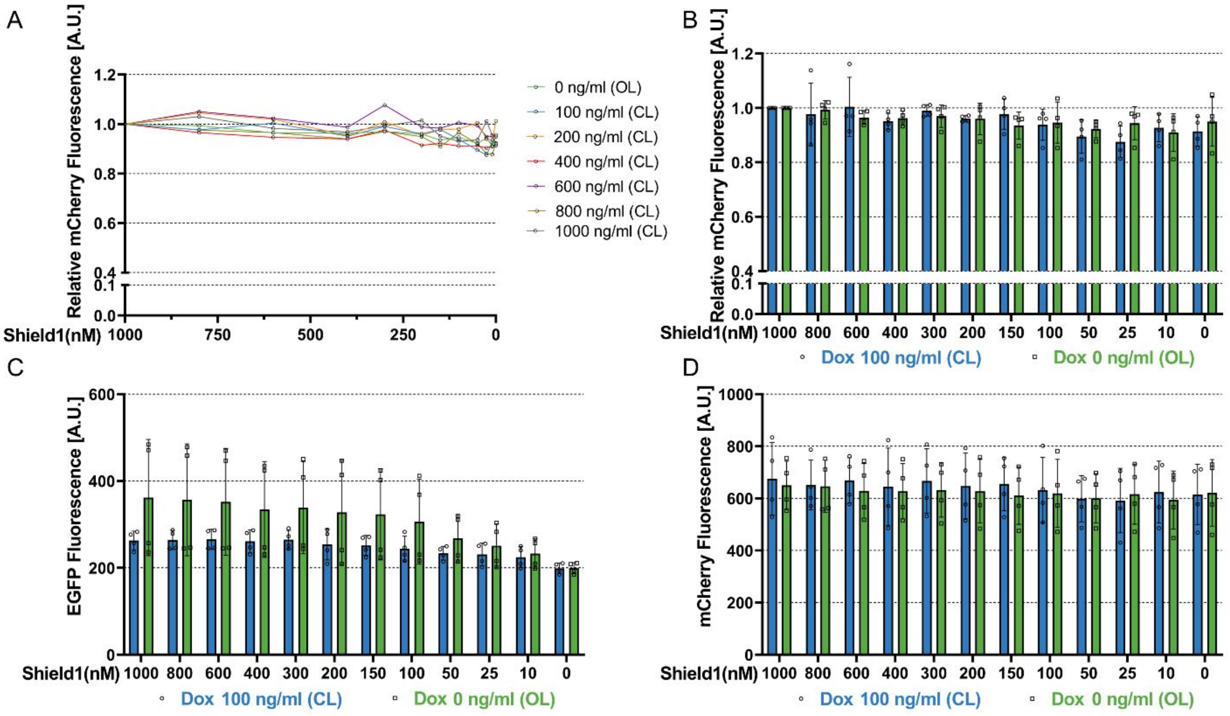

Figure S4 – Robust Perfect Adaptation in stable cell lines: A) Quantification of the red fluorescence (mCherry) of Hek293T cells integrated with the CRISPRaTOR for decreasing concentrations of Shield1 and for the indicated fixed concentration of Doxycycline. In the absence of Doxycycline, the CRISPRaTOR is in the Open Loop configuration. Fluorescence values are relative to the value measured at 1000 nM Shield1. **B)** Relative red fluorescence measured in (A) but represented as a bar plot for only two conditions: Doxy 100 ng/ml (blue) and without Doxycycline (green). **C, D)** Absolute measurement values of the green fluorescence (C) and the red fluorescence (D) for the experiment in Figure 3d. *For imaging quantification experiments, n=4 biological replicates coming from 4x96 well plates.*
